# *Dermocystidium* sp. infection in farmed striped catfish *Pangasianodon hypophthalmus* farmed in Ceará state, Northeastern Brazil

**DOI:** 10.1590/S1984-29612022025

**Published:** 2022-05-27

**Authors:** Pedro Henrique Magalhães Cardoso, Rachel Sordi Relvas, Simone de Carvalho Balian, Andrea Micke Moreno, Herbert Sousa Soares, Luiz Augusto Santana Silva, Maurício Laterça Martins

**Affiliations:** 1 Departamento de Medicina Veterinária Preventiva, Faculdade de Medicina Veterinária e Zootecnia, Universidade de São Paulo – USP, São Paulo, SP, Brasil; 2 Programa de Pós-Graduação em Medicina Veterinária e Bem Estar Animal e Saúde Única, Universidade Santo Amaro – UNISA, São Paulo, SP, Brasil; 3 Laboratório Vet Fácil, São Paulo, SP, Brasil; 4 Laboratório de Sanidade de Organismos Aquáticos – AQUOS, Departamento de Aquicultura, Universidade Federal de Santa Catarina – UFSC, Florianópolis, SC, Brasil

**Keywords:** Dermocystidium, pangasius, Pangasianodon hypophthalmus, histopathology, Dermocystidium, pangasius, Pangasianodon hypophthalmus, histopatologia

## Abstract

The genus *Dermocystidium* infects a wide range of animals. The host infection often occurs through the ingestion of endospores. The diagnosis depends on wet mounts and histopathological analysis of the affected tissue. The aim of this study was to investigate the incidence of *Dermocystidium* sp. infection on the skin of farmed striped catfish (*Pangasianodon hypophthalmus*) from a fish farm located in Fortaleza, Ceará state, northeastern Brazil. From these observations, we determined that 100% of the analyzed animals were infected with *Dermocystidium* sp. The wet mount and histopathology of the fish lesions revealed spore-filled cysts between the dermis and epidermis, encapsulated by connective tissue. Owing to a lack of research on the parasite and its prevalence among different fish species in Brazil and the rest of the world, additional studies are required to understand their endemicity in fish farms of Brazil, and consequently develop better disease prevention methods and increase the overall productivity.

## Resumo

O gênero *Dermocystidium* infecta uma ampla variedade de espécies de animais, e o hospedeiro pode se infectar pela ingestão de endósporos. O diagnóstico depende de montagem em lâmina a fresco e análise histopatológica dos tecidos afetados. O objetivo deste estudo foi relatar infecção por *Dermocystidium* na pele do bagre listrado (*P. hypophthalmus*), cultivado em piscicultura do estado do Ceará, nordeste do Brasil. Foi detectado que 100% dos animais analisados estavam infectados por *Dermocystidium* sp. A montagem em lâminas a fresco e histopatologia das lesões mostrou cistos repletos de esporos entre a derme e a epiderme, encapsulados por tecidos conectivos. Devido às escassas pesquisas sobre esse parasito, em diferentes espécies de peixes no Brasil e no mundo, estudos adicionais são necessários para entender a endemicidade em diferentes pisciculturas do Brasil, consequentemente, melhorando os métodos de prevenção da doença e aumentando a produção e a produtividade.

A spike in prices in the global pangasius market in 2018 caused an increase in the production of fish species (FAO, 2020). In 2019, the species gained importance in northeastern (Piauí, Maranhão, and Rio Grande do Norte states) and southeastern Brazil (especially São Paulo state); in Rio Grande do Norte state, there was a 32.8% growth in the total fish production, compared to 2018, with pangasius representing more than 40% of the fish farm production in 2019 (Carvalho et al., 2020).

Fish may become stressed due to stock management practices under conditions employed in the intensive aquaculture systems, leading to immunosuppression and susceptibility to parasitic infections (Jerônimo et al., 2012).

*Dermocystidium* belongs to the class Mesomycetozoea. Despite being classified as protists, they exhibit features from both the Protista and Fungi kingdoms (Mendoza et al., 2002). They infect a wide range of animals (both vertebrates and invertebrates), such as amphibians, molluscs, and a variety of bony fishes such as cyprinids, salmonids, cichlids, eels, lampreys, and catfishes (Ray & Chandler, 1955; Mendoza et al., 2002; Zhang & Wang, 2005; Bruno et al., 2006; Mahboub & Shaheen, 2020; Sellyei et al., 2020).

Water pollution (Valtonen et al., 2003) and thermal stress, especially at lower temperatures (Zhang & Wang, 2005; Mahboub & Shaheen, 2020), may act as predisposing factors to *Dermocystidium* infection. This infection manifests macroscopically as small spherical, oval, or elongated white nodules or cysts (sporangia) located in the epithelial tissue of the skin, fins, gills, and eyes or internal organs (chronic systemic infections) (Eiras & Silva-Souza, 2000; Mendoza et al., 2002; Zhang & Wang, 2005; Bruno et al., 2006; Mahboub & Shaheen, 2020).

The host can be infected by direct transmission through ingestion of the endospores (for example, ingestion of infected fish) or through water by the attachment of endospores to the gills and skin (Mendoza et al., 2002; Mahboub & Shaheen, 2020). Within the host, the parasite forms hyaline cysts (Mendoza et al., 2002). Diagnosis is contingent on wet mounts and histopathological analysis of the affected tissue (Eiras & Silva-Souza, 2000; Zhang & Wang, 2005; Bruno et al., 2006; Fujimoto et al., 2018). The latter displays unicellular spherical spores with a solid refractile body (skin and gill infections) or a large central vacuole (systemic infections) (Bruno et al., 2006). Recently, Mahboub & Shaheen (2020) discovered another sensitive and effective diagnostic method by isolating *Dermocystidium* from recently deceased fish. It consists of culture on Tris-buffered Eagle’s Minimum Essential Medium (MEM) at pH 3.5 and, subsequently, on Sabouraud dextrose agar (SDA) with chloramphenicol and 10% duck decoction.

In Brazil, *Dermocystidium* infections have been reported in wild catfish (*Trichomycterus* sp.) (Eiras & Silva-Souza, 2000), tambatinga, a hybrid of *Colossoma macropomum* and *Piaractus brachypomus* (Fujimoto et al., 2018), Nile tilapia, *Oreochromis niloticus* (Steckert et al., 2019), and channel catfish, *Ictalurus punctatus* (Relvas et al., 2020).

**Figure 1:**
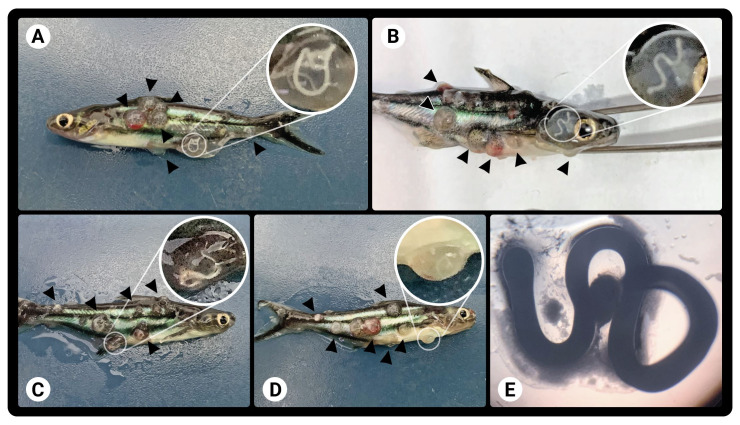
(A-D) Striped catfish (*Pangasianodon hypophthalmus*) with cysts lesions in the skin and; (E) and the wet mount of the cyst (optical magnification 4x).

The study aimed to investigate *Dermocystidium* sp. infection on the skin of *P. hypophthalmus* from a fish farm from Fortaleza, in Ceará state, northeastern Brazil. Ten striped *P. hypophthalmus* **(**± 3.6 g weight and ± 7.55 cm long) with clinical signs of skin lesions (Figure 1A-D) were obtained from a fish farmer in February 2021. On the farm, fish were stocked at 2000 fish/m^3^. There was no quarantine routine before introducing the new fish into the general population. For analysis, the fish were transported in plastic bags to São Paulo City, southeastern Brazil. They were then classified based on their weight and total length. The fish were rapidly sedated by treating with eugenol (100 mg L^-1^ of clove oil) for 3 min (Roubach et al., 2005) and euthanized by medullar section (Noga, 2010). Fish were dissected, and the skin and other affected tissues were sampled for diagnostic purposes.

Cysts were removed from the fish skin, and wet mounts were performed using a Zeiss light microscope at 4 ×, 10 ×, 20 ×, and 40 × objective magnification. Parasites were identified based on their structure and morphology. The affected skin tissue and other internal organs such as the liver, spleen, stomach, and intestine of the infected fish were fixed in 10% phosphate-buffered formalin, embedded in paraffin, and sectioned with hematoxylin and eosin stain (H&E).

**Figure 2:**
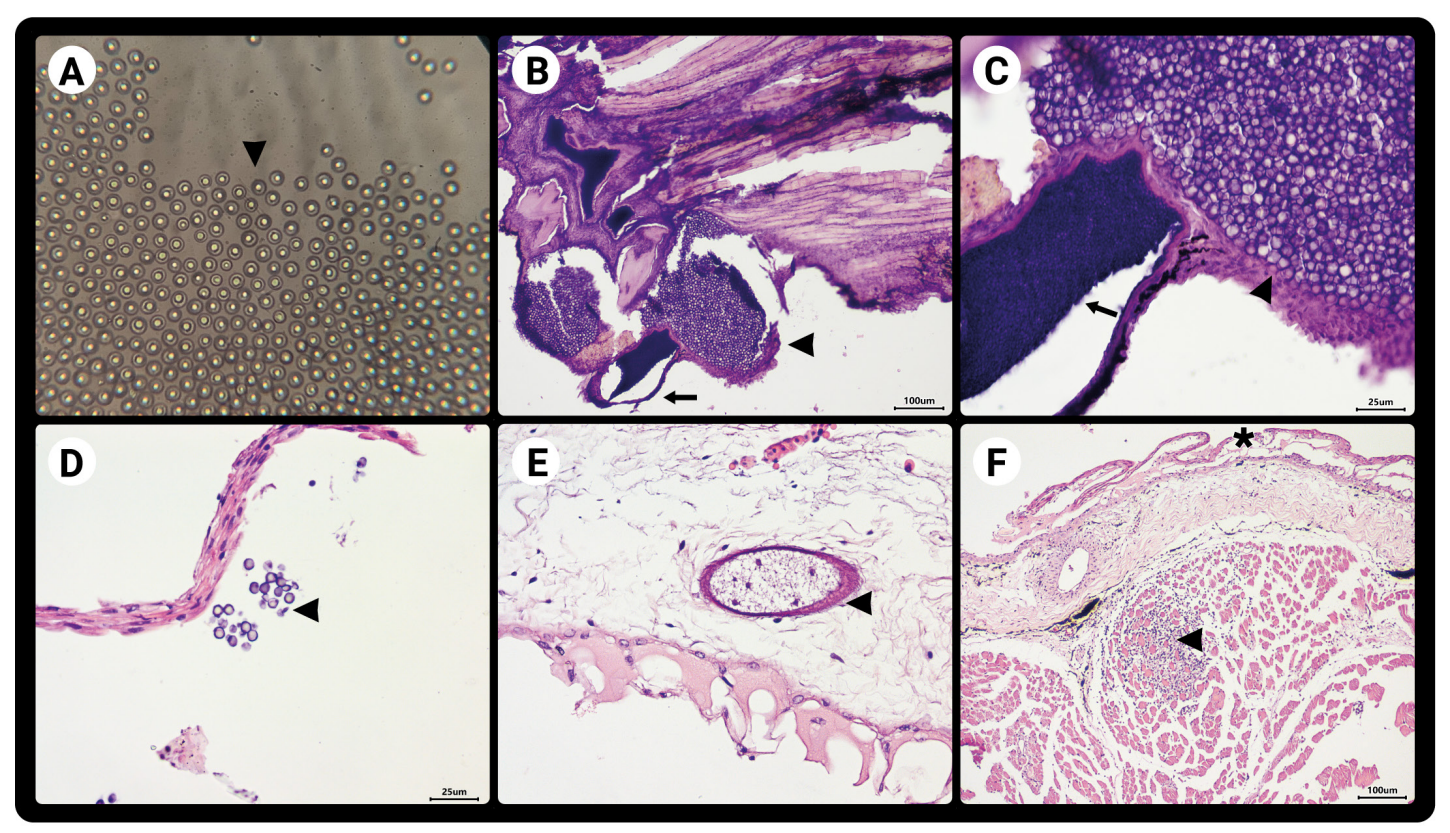
(A) Spores in the slide (wet mount optical magnification 40x); (B-F) Histological skin sections stained with hematoxylin and eosin. (B) Cutaneous section showing a spore-rich cyst (arrow) and a large amount of free spores (arrowhead), (C) Histological section at higher magnification of figure 'B' in 40x optical magnification; (C) There is a cyst in the lower right corner rich in spores and a large amount of free spores; (D) Small amount of free spores, in 40x optical magnification; (E) Histological section of the skin showing a cyst in the dermis that is poor in spores; (F) Histological section of the skin revealing marked vacuolization of the epidermis, with formation of vesicles and an inflammatory process located in the muscle tissue.

Wet mount observations revealed that 100% of the fish were infected with *Dermocystidium* sp. The wet mount and histopathology of their lesions showed spore-filled cysts (Figure 1E and Figure 2A) between the dermis and epidermis, encapsulated by connective tissue (Figure 2B-E). Histological sections of the skin revealed vacuolization of the epidermis, with the formation of vesicles and an inflammatory process in the muscle tissue (Figure 2F). No significant histopathological changes were observed in any of the internal organs.

*Dermocystidium* sp. belongs to a class of organisms, the Mesomycetozoea, which exists between the protozoan and metazoan boundaries. Despite being classified as protists, they also exhibit features from the fungal kingdom (Mendoza et al., 2002). Infected fish display external cysts (spherical sporangia) on their skin, gills, eyes, or internal organs, while systemic infections are less frequent (Eiras & Silva-Souza, 2000; Mendoza et al., 2002; Zhang & Wang, 2005; Mahboub & Shaheen, 2020). Zhang & Wang (2005) reported that juvenile fishes are more susceptible to infection, corroborating our results. A correlation exists between infections of *Dermocystidium* and thermal stress in *Silurus meridionalis* catfish. Mahboub & Shaheen (2020) reported the highest infection rates in *O. niloticus* during winter. In the case study, the occurrence was in the summer.

There are four incidents of this parasite reported in Brazil. The first was seen in wild catfish (*Trichomycterus* sp.) (Eiras & Silva-Souza, 2000) in Paraná, the second in tambatingas (Fujimoto et al., 2018) in Sergipe, the third in Nile tilapia (*O. niloticus)* in Santa Catarina (Steckert et al., 2019) and the fourth in channel catfish (*I. punctatus)* in Minas Gerais (Relvas et al., 2020). The clinical signs and histological sections of *P. hypophthalmus* analyzed in our study suggest a *Dermocystidium* sp. infection. Therefore, this is the first report of *Dermocystidium* in striped catfish, *P. hypophthalmus*, in Brazil.

Considering the economic value of pangasius and the possible route of direct transmission, *Dermocystidium* spp. infections should be considered an important disease in this species. Owing to the lack of research on the *Dermocystidium* parasite and its presence in different fish species in Brazil, additional studies are needed to understand the endemicity of the parasite in different regions and the fish farms of Brazil, and consequently, devise better methods of disease prevention and increase overall productivity. Molecular analyses are also necessary to determine differences between parasites in different fish species.
